# Ryanodine Receptor 1-Related Myopathies: Diagnostic and Therapeutic Approaches

**DOI:** 10.1007/s13311-018-00677-1

**Published:** 2018-11-07

**Authors:** Tokunbor A. Lawal, Joshua J. Todd, Katherine G. Meilleur

**Affiliations:** Neuromuscular Symptoms Unit, National Institute of Nursing Research, National Institutes of Health, Bethesda, MD USA

**Keywords:** *RYR1*, Myopathies, Therapeutics, Central core disease, N-acetylcysteine, Rycal, 4PBA, Salbutamol

## Abstract

Ryanodine receptor type 1-related myopathies (*RYR1*-RM) are the most common class of congenital myopathies. Historically, *RYR1*-RM classification and diagnosis have been guided by histopathologic findings on muscle biopsy. Main histological subtypes of *RYR1*-RM include central core disease, multiminicore disease, core–rod myopathy, centronuclear myopathy, and congenital fiber-type disproportion. A range of *RYR1*-RM clinical phenotypes has also emerged more recently and includes King Denborough syndrome, *RYR1* rhabdomyolysis-myalgia syndrome, atypical periodic paralysis, congenital neuromuscular disease with uniform type 1 fibers, and late-onset axial myopathy. This expansion of the *RYR1*-RM disease spectrum is due, in part, to implementation of next-generation sequencing methods, which include the entire *RYR1* coding sequence rather than being restricted to hotspot regions. These methods enhance diagnostic capabilities, especially given historic limitations of histopathologic and clinical overlap across *RYR1*-RM. Both dominant and recessive modes of inheritance have been documented, with the latter typically associated with a more severe clinical phenotype. As with all congenital myopathies, no FDA-approved treatments exist to date. Here, we review histopathologic, clinical, imaging, and genetic diagnostic features of the main *RYR1*-RM subtypes. We also discuss the current state of treatments and focus on disease-modulating (nongenetic) therapeutic strategies under development for *RYR1*-RM. Finally, perspectives for future approaches to treatment development are broached.

## Introduction

Congenital myopathies (CM) are an expanding group of genetic disorders typically characterized by childhood onset of skeletal muscle hypotonia and slowly or nonprogressive skeletal muscle weakness [1]. The collective true prevalence of CM is not well understood. Regional estimates range from 1:26,000 (pediatric) in the USA to 1:135,000 in the UK (pediatric and adult) [2–5]. Of all CM, ryanodine receptor 1-related myopathies (*RYR1*-RM), with an estimated US pediatric point prevalence of at least 1:90,000, are recognized as the most frequently diagnosed. This is in part owing to advances in genetic testing [6, 7].

The *RYR1* gene (19q 13.2) encodes a calcium ion (Ca^2+^) channel (RyR1), which is embedded within the sarcoplasmic reticulum (SR) membrane in skeletal muscle. This large gene spans > 159 kb and features an open reading frame encoded in 106 exons (2 alternatively spliced) [8]. RyR1 is a homotetrameric protein structure comprised of 4 identical ~ 560-kDa subunits, each of which houses an FK506-binding protein (FKBP) subunit. The RyR1-FKBP interaction is crucial for channel stability, as are accessory proteins such as calmodulin, triadin, junctin, and calsequestrin [9]. *RYR1*-RM encompass a heterogeneous spectrum of histopathological and clinical subtypes accompanied by varying disease severity.

Histopathological *RYR1*-RM subtypes include central core disease (CCD) [10, 11], multiminicore disease (MmD) [12], centronuclear myopathy (CNM) [13], core–rod myopathy (CRM) [14], and congenital fiber-type disproportion (CFTD) [15]. Clinical phenotypes resulting from *RYR1* variants are diverse and include interrelated conditions such as malignant hyperthermia (MH) susceptibility, exertional heat stroke, rhabdomyolysis-myalgia syndrome, King Denborough syndrome, and atypical periodic paralysis [5, 16, 17].

This review focuses on disease-modulating (nongenetic) therapeutic strategies under development for *RYR1*-RM. An overview of *RYR1*-RM diagnostic features [18] and potential therapeutic approaches are discussed.

## Genetic Etiology

Historically, dominant *RYR1* variants have been enriched in 3 hotspot regions (N-terminal: MH/CCD region 1, amino acids 35–614; central: MH/CCD region 2, amino acids 2163–2458; and C-terminal: MH/CCD region 3, amino acids 4550–4940), with region 1 and 2 variants predominantly associated with the MH susceptibility phenotype and region 3 variants with the classic CCD phenotype. *RYR1* variants attributed to a recessive mode of inheritance have been reported as being evenly distributed throughout the *RYR1* sequence [19–21]. Dominantly inherited *RYR1* variants have been most frequently linked to CCD and MH susceptibility, whereas recessively inherited variants are prevalent in individuals with MmD [19], CNM [22], and CFTD histopathology [15].

MH (MIM# 145600) is a potentially lethal hypermetabolic condition triggered by exposure of susceptible individuals to certain volatile anesthetics and muscle relaxants. MH-like events triggered by nonpharmacologic factors (high environmental and body temperature, stress) have also been reported [23, 24]. With incomplete penetrance of MH susceptibility (~ 50%) and no common *RYR1* causative variant, the diagnostic test for MH susceptibility is the *in vitro* caffeine–halothane contracture test (IVCT), which determines contracture thresholds. The IVCT requires an open biopsy to obtain sufficient fresh skeletal muscle tissue and may yield false-negative results [25]. Approximately 50% of MH-susceptible individuals exhibit cores on biopsy whereas others, without a history of myopathy prior to an MH episode (MH trait only), do not [8, 26, 27]. It is therefore challenging to rule out MH susceptibility in individuals with pathogenic *RYR1* missense variants, and this is compounded by the fact that susceptible individuals do not always exhibit symptoms following initial exposure(s) to triggering agents. Furthermore, although MH susceptibility is typically associated with dominant *RYR1*-RM cases, MH episodes have been reported in recessive cases [28]. As such, all *RYR1*-RM-affected individuals are considered potentially MH susceptible [29].

Once a clinical phenotype of CM is suspected, histopathological findings such as cores, rods, centralized nuclei, and type 1 fiber predominance on skeletal muscle biopsy provide evidence for classification of *RYR1*-RM subtypes. Muscle imaging (magnetic resonance imaging and ultrasound) has become utilized more recently as part of the differential diagnosis. Muscle imaging has proven particularly useful for *RYR1-*RM owing to a specific pattern of muscle involvement that is often visible on MRI and ultrasound, namely relative sparing of the rectus femoris compared with the vastus lateralis [30].

Advancements in next-generation sequencing (NGS) technologies have made it possible to screen for variants in large genes such as *RYR1* and assess genotype–phenotype correlations [31, 32]. *RYR1* variants have been implicated in sarcoplasmic reticulum (SR) calcium Ca^2+^ release dysfunction (RyR1 channel hyper/hyposensitivity and chronic Ca^2+^ leak) [33]. Recessive variants have also been associated with hypomorphic RyR1 protein expression and RyR1-dihydropyridine (DHPR) misalignment [33, 34]. However, further research is required in order to fully elucidate the complex pathomechanism(s) elicited by specific *RYR1* variants with regard to channel and calcium regulation.

As is the case with other congenital myopathies, such as *SEPN1*-related myopathy (*SEPN1*-RM), another core myopathy, no FDA-approved treatments are available to date. Nevertheless, there have been reports of off-label drug responses to manage related symptoms, which we discuss below under “*RYR1*-RM Therapeutic Approaches” [7, 9, 35–37].

## Ryanodine Receptor Isoform 1

Ryanodine receptor isoform 1 (RyR1) is the major Ca^2+^ channel expressed in skeletal muscle and is a critical component of the triad system that is required for effective excitation–contraction (EC) coupling. RyR1 are embedded in the membranes of sarco/endoplasmic reticula (SR/ER) and regulate the rapid intracellular release of Ca^2+^ following transverse tubule depolarization. RyR isoforms also contribute to maintaining cellular Ca^2+^ homeostasis under resting conditions [38]. With a total molecular weight of ~ 2200 kDa, these homotetrameric membrane proteins are the largest known ion channels, capable of raising cytosolic Ca^2+^ by 2 orders of magnitude in < 0.5 ms [38, 39]. There are 2 additional mammalian RyR isoforms (RyR2 and RyR3) for which expression has been detected in numerous tissues and cell types, including cardiac muscle (where RyR2 is the predominant isoform), brain, smooth muscle, exocrine cells, epithelial cells, dendritic cells, and B-lymphocytes [9, 40, 41]. There is approximately 65% homology between isoforms [42]; however, RyR1 was the first to be cloned [43] and extensively described using both cryo-electron microscopy (cryo-EM) [44] and X-ray crystallography of individual domains [45]. RyR1 serves as a connection between the SR and the transverse tubule (invaginations of the sarcolemma) in skeletal muscle, allowing for direct protein–protein coupling to the L-type Ca^2+^ channel dihydropyridine (DHPR) receptors that are localized to the transverse tubule membrane. Depolarization across the plasma membrane causes conformational changes in DHPR that trigger RyR1 opening independently of extracellular Ca^2+^ concentration [46].

Aberrant SR Ca^2+^ release leading to disruption of cytosolic Ca^2+^ homeostasis is the direct consequence of RyR1 dysfunction [33, 47]. Consequently, EC uncoupling between RyR1 and DHPR, RyR1 channel destabilization causing SR Ca^2+^ leak which is ineffectively compensated by the sarco-endoplasmic reticulum Ca^2+^ ATPase (SERCA) pump (decompensated leak), decreased RyR1 expression, and elevated oxidative and nitrative stress resulting from mitochondrial Ca^2+^ uptake dysregulation could explain the phenotypes associated with *RYR1*-RM [48–51].

## *RYR1*-RM Diagnostic Approaches

A summary of diagnostic clues suggestive of *RYR1*-RM is presented in Table 1.

**Table 1:** Diagnostic clues suggestive of RYR1-RM

	Central core disease	Multiminicore disease	Core–rod myopathy	Centronuclear myopathy	Congenital fiber type disproportion
Histopathology	Single or multiple cores spanning the longitudinal fiber axis	Multiple cores; limited on longitudinal sections	Presence of both central cores and nemaline bodies upon muscle biopsy	Centrally located nuclei	Fiber size disproportion (type 1 fibers consistently at least 35–40% smaller than type 2 fibers diameter)
	Type 1 fiber predominance and uniformity	Type 1 fiber predominance and uniformity		Type 1 fiber predominance and uniformity	Type 1 fiber predominance
	Increased internal nuclei			Type 1 hypotrophy	
				Central aggregation or reduction of oxidative stains	
				Cores on oxidative stains	
Clinical features	Hypotonia and motor development delay	Hypotonia	Axial hypotonia		Static or slowly progressive generalized muscle weakness
	Respiratory, bulbar, and cardiac involvement are uncommon	Early respiratory impairment with or without cardiac complications	Respiratory impairment	Mild respiratory involvement	Respiratory weakness
	Proximal weakness pronounced in hip girdle	Distal weakness	Diffuse muscle weakness	Diffuse and progressive muscle weakness	Proximal axial weakness
		Extraocular muscle involvement and ophthalmoplegia in severe cases		Ptosis/extraocular involvement	Ophthalmoplegia
			Mild facial involvement	Facial dysmorphism	Facial muscle weakness
	Orthopedic deformities (scoliosis) and ligamentous laxity	Spinal rigidity and scoliosis	Multiple joint contractures and scoliosis	Joint contractures	
		Moderate bulbar involvement		Bulbar weakness	Dysphagia
	Myalgia and/or exertional weakness with or without rhabdomyolysis	Exercise-induced myalgia			
	High malignant hyperthermia susceptibility	Malignant hyperthermia rarely reported			

## Genetic Testing

Although genotype–phenotype correlations remain challenging to establish across subtypes of this disease, the significant overlap and common occurrence of histologic features in the *RYR1*-RM spectrum make it increasingly likely that associated disorders will ultimately be defined by genetics and pathophysiology, and less by histopathology [1, 52]. *RYR1*-RM genetic heterogeneity and the increasing utility of NGS approaches to variant identification, coupled with reduction in sequencing cost, should prompt clinicians and researchers to screen patients for pathogenic variants with congenital myopathy sequencing panels over single gene analysis [53, 54]. Indeed, genetic sequencing (by gene, targeted variant, exome, and/or genomic testing) is becoming an increasingly crucial diagnostic tool for *RYR1*-RM and sequencing the entire *RYR1* gene rather than only the 3 hotspots is now best practice.

Muscle imaging, including both magnetic resonance imaging and ultrasound, has also become utilized as an adjunct diagnostic tool. A specific pattern of muscle involvement in *RYR1*-RM is typically evident on MRI and ultrasound [30]. The combination of neuromuscular panel genetic testing and muscle imaging facilitates diagnosis in many cases, avoiding an invasive biopsy and the use of anesthesia in this malignant hyperthermia-susceptible population. If genetic testing and muscle imaging are inconclusive, biopsy may then be performed to further direct a diagnosis. Muscle imaging is discussed further below.

## Clinical Phenotypes Indicative of *RYR1*-RM

Clinical features associated with *RYR1*-RM have been extensively described [1, 16, 20]. Across all ages, *RYR1*-RM clinical features include symmetric proximal muscle weakness, often with pronounced facial weakness with or without dysmorphism and ophthalmoparesis/ophthalmoplegia with ptosis, bulbar weakness, significant respiratory involvement, severe neonatal hypotonia, scoliosis, orthopedic deformities including arthrogryposis, hip dislocation, club feet, and King Denborough syndrome (pectus carinatum or excavatum, short stature, joint contractures, facial and skeletal deformities) [16], MH susceptibility, anesthesia-induced rhabdomyolysis [55], fatigue, exercise-induced hyperthermia/exertional heat stroke, and exertional myalgia [56].

## Histopathology, Clinical Features, and Genetic Considerations

### Central Core Disease

Histopathologic features: On oxidative histological staining, CCD presents with clearly demarcated central cores (regions of pale staining) that can span the length of the muscle fiber. The amorphous central cores predominate within type 1 fibers and result from the absence or depletion of mitochondria [8]. These patterns show variability ranging from more than 1 core per muscle fiber, variable core diameters, to typical or eccentric cores and centrally or peripherally located cores [57]. Sarcomeric filamentous disorganization (Z line streaming) and proliferation of sarcotubular membranes are typical on electron microscopy [58].

Clinical features: As one of the most frequent CM [59], the CCD phenotype of dominant *RYR1* causative variations is usually mild and nonprogressive. Clinical features include hypotonia in infancy, delayed motor milestones, axial muscle involvement, proximal weakness, myalgia, muscle stiffness, hip dislocation, spinal deformities, and exertional weakness with or without rhabdomyolysis. Extraocular muscle weakness and bulbar and respiratory involvement are not typical [33, 60]; however, strabismus has been reported in dominant cases [31, 61]. Recessively inherited or *de novo* dominant *RYR1*-related CCD present with more severe features such as fetal akinesia syndrome (severe hypotonia, multiple arthrogryposis, respiratory failure) [8, 61, 62]. Serum creatinine kinase (CK) may be mildly elevated or normal, and muscle MRI imaging shows selective involvement and sparing of certain muscle groups in dominant *RYR1*-related cases [30]. MRI findings in recessively inherited cases are not as consistent. There is an overlap between CCD and MH susceptibility, with *RYR1*-related CCD patients with causative variants in the N-terminal region having a higher probability of MH susceptibility than CCD patients with variations in the C-terminal region [8].

Genetic considerations: Approximately 60% of *RYR1*-related CCD causative variants are located in the CCD hotspots including the C-terminal domain. CCD is usually inherited in an autosomal dominant pattern with few recessively inherited cases [61]; therefore, parental genetic testing should be performed when possible. To increase the probability of detecting a causative variant, individuals suspected of *RYR1*-RM should have full *RYR1* gene sequencing performed [8, 63]. A CCD patient, with an asymptomatic parent who tests positive for the same variant, should be investigated for additional *RYR1* variants by sequence analysis of the entire *RYR1* gene to investigate the possibility of recessive modes of inheritance. More than half of the variants associated with *RYR1*-RM are private, and only 10% or so of *RYR1* variants have been functionally characterized. Therefore, additional variants identified through genetic testing may have unknown or unreported functions (variants of unknown clinical significance) [29]. Variations in the *MYH7* gene are increasingly associated with CCD histopathology, making up an estimated 10% of CCD cases [64]. Other genes implicated in CCD histopathology include *SEPN1*, *ACTA1*, *TTN*, *KBTBD13*, and *CCDC78*, *CACNA1S* [7, 18, 65].

### Multiminicore Disease

Histopathologic features: MmD is a recessive *RYR1*-RM subtype that presents with numerous amorphous cores on oxidative muscle biopsy stains, resulting in a characteristic “moth-eaten” appearance. The cores often vary in size, are observed in most fibers, and typically do not span the entire length of the muscle fiber, unlike those in CCD. Multiple internally placed nuclei and type 1 fiber predominance are also noted in affected muscles. Minimal disruption of myofibrillar structure may be present on electron microscopy. Abnormal expression of desmin and other sarcomeric and intermediate filament proteins may be observed within or around the core area in immunohistochemical studies.

Clinical features: As with most *RYR1*-RM subtypes, MmD shows clinical diversity and genetic heterogeneity. Clinical features in MmD patients include delayed development, proximal and axial weakness, hip girdle involvement, marked distal weakness and wasting, feeding difficulty during infancy, prominent facial weakness, axial hypotonia, scoliosis, ophthalmoplegia, and severe respiratory impairment [66]. Homozygosity or compound heterozygosity for either 2 missense variants or 1 nonsense and 1 missense variant is the most common mode of inheritance in recessive *RYR1*-related MmD [66, 67]. The clinical phenotype of recessively inherited *RYR1*-associated MmD may be partly explained by decreased RyR1 protein expression in affected individuals [33].

Genetic considerations: *RYR1* should be considered the second most likely causative gene to *SEPN1* for MmD [18]. *SEPN1* encodes selenoprotein-N, which is involved in cellular redox-regulated calcium homeostasis. *SEPN1*-related MmD is characterized by early spinal rigidity, high-pitched voice, myopathic facies, respiratory involvement, and scoliosis, and does not typically present with extraocular muscle involvement and ophthalmoplegia [18, 60]. If cardiomyopathy is present, variants in *TTN* and *MYH7* genes should be considered [68]. Variations in *MEGF10* and *CACNA1S* have also been associated with MmD [65, 69]. Screening of the entire *RYR1* coding sequence as part of a congenital myopathy sequencing panel is recommended to effectively elucidate the genetic etiology of MmD histopathology.

### Core–Rod Myopathy

Histopathologic features: CRM can be identified by the presence of both central cores and nemaline bodies (rods), characteristic of nemaline myopathy (MIM# 256030), upon biopsy. *RYR1* variants are the most common cause of CRM, although causative variants in other genes have been reported [70, 71]. Rods are largely composed of actin and α-actinin with the same electron density similar to the Z lines of adjacent sarcomeres on EM. Rods are arranged in clusters or diffusely distributed within the fibers. Structured cores spanning almost the entire fiber length with absent mitochondria are seen in longitudinal sections. CRM myopathic features include centralized nuclei, variability in fiber size diameter, type 1 fiber predominance, and mild fatty infiltration. Single and multiple cores, centrally and peripherally located, coexist with rods [14].

Clinical features: Patients present with generalized hypotonia, mild facial weakness, diffuse muscle weakness with axial predominance, multiple joint contractures, scoliosis, and respiratory insufficiency. Serum CK levels are normal and cardiac complications are typically absent.

Genetic considerations: Variants in *KTBTBD13* and *CFL2* genes have been associated with CRM. *KTBTBD13* encodes a muscle-specific ubiquitin ligase, and variants in this gene can cause both CRM and nemaline myopathy. *KTBTBD13* variants should be suspected in CRM patients with proximal weakness and “slowness” in muscle movement [18]. Compound heterozygous cases with *NEB* variants have also been associated with CRM [71, 72]. Additionally, variations in genes associated with nemaline myopathy (*NEM1*, *ACTA1*, *TPM3*) should be considered and screened as part of a NGS congenital myopathy sequencing panel to identify causative variants.

### Centronuclear Myopathy

Histopathologic features: CNM is characterized by numerous centrally located nuclei in approximately ≥ 25% of muscle fibers [73]. The number of centralized nuclei may increase with age, confounding the minimum number required for diagnosis [74]. Central and multiple internalized nuclei are the main pathological features noted when muscle biopsy is performed in early life, but other histopathological features associated with recessive *RYR1*-RM appear over time [75]. Histopathological features include a central zone with either accumulation or absence of oxidative enzyme activity on NADH staining, radial strands (myofibrils) surrounding the central zone in many fibers, variability in fiber diameter, and type 1 fiber predominance and hypotrophy [33].

Clinical features: CNM is part of the recessive *RYR1*-related continuum, and therefore, patients present with substantial clinical similarities as noted with MmD. Clinical presentations include facial weakness, external ophthalmoplegia, and predominantly proximal muscle involvement [22]. Variants in multiple genes encoding proteins linked to various aspects of phosphoinositide metabolism and membrane trafficking, T-tubule formation, triad assembly, and EC coupling (*DNM2*, *TTN*, *BIN1*, *MTMR14*, *CCDC78*) [76] have been associated with CNM, including the most severe fatal X-linked form (myotubular myopathy) attributed to mutations in the *MTM1* gene [77]. Most frequent clinical features of autosomal CNM include delayed motor milestones, distal muscle weakness, ptosis, ophthalmoparesis, or ophthalmoplegia [78]. More recently, late-onset distal myopathy has also been associated with *RYR1*-CNM [79].

Genetic considerations: Caution should be taken to exclude recessive inheritance if only a single variant is identified, especially as *RYR1* is a large gene and has many variants of unknown significance. Screening of the entire *RYR1* coding sequence as part of a congenital myopathy sequencing panel is recommended in identification of CNM causative variants. In severely affected males, the investigative focus should begin with variants in the *MTM1* gene, including deep intronic regions to capture rare variants, for the X-linked form of CNM. Female carriers of *MTM1* variants with moderate to severe symptoms may have skewed X-inactivation [75]. Variations in *DNM2* gene should be investigated if family history and clinical features are consistent with autosomal dominant inheritance and predominant distal weakness [74, 78, 79].

### Congenital Fiber-Type Disproportion

Histopathological features: CFTD histopathological diagnosis requires observation of type 1 fibers that are 35–40% consistently smaller than type 2 fibers in the absence of other structural abnormalities [80]. This disproportion has to be the main diagnostic abnormality after ruling out other histopathological findings for the diagnosis of CFTD to hold. When fiber size disproportion appears in conjunction with rods, cores, or central nuclei, it is superseded by those histopathological findings. *RYR1* variants may account for up to 20% of CFTD cases, while other well-established genetic etiologies include variations in *ACTA1*, *TPM3*, *TPM2*, *SEPN1*, and *MYH7* genes [15, 81]. No single clinical or histologic feature is specific to CFTD; therefore, clinical features should be consistent with congenital myopathies to make a CFTD diagnosis.

Congenital neuromuscular disease with uniform type 1 fiber (CNMDU1) is associated with variations in the C-terminal domain of *RYR1*, a hotspot region for CCD causative variants. CNMDU1 is a rare form of CM that is pathologically diagnosed by a muscle fiber composition of > 99% type 1 fibers with no other specific structural changes [82]. The few type 2 fibers present are predominantly immature type 2C.

Clinical features: Overlapping with other recessive *RYR1*-RM, *RYR1*-related CFTD clinical presentations include prominent hypotonia and weakness of axial muscles, myopathic facies, respiratory failure, feeding difficulties, and ophthalmoparesis. Joint contractures, scoliosis, and cardiac involvement are relatively uncommon. Muscle imaging of the leg typically shows sparing of the rectus femoris compared with other quadriceps muscles [83]. Cases of *RYR1*-CNMDU1 present with clinical features similar to other CM such as infantile hypotonia, mild proximal muscle weakness, respiratory distress, high arched palate, craniofacial dysmorphism, normal CK levels, and no mental retardation.

Genetic considerations: Known genes account for 50 to 70% of causative variations in CFTD; therefore, other genetic causes remain unidentified [84]. Variants in *TPM3* and *ACTA1* genes should be investigated if an autosomal dominant inheritance pattern is suspected. Identification of causative variations in particular genes can determine which disease surveillance is recommended, for example, cardiac surveillance in *MYH7* and *TMP2* pathogenic variations [80]. Heterozygous *RYR1* sequence variations (2 missense, 1 substitution of 2 consecutive nucleotides, and 1 *de novo* frame-shift deletion) have been reported in CNMDU1 patients [82]. However, the genetic etiology and pathomechanism of the CNMDU1 remain uncertain. Exome sequencing or NGS congenital myopathy sequencing panel is recommended for detection of possible causative variants in individuals affected by CFTD and CNMDU1.

## Magnetic Resonance and Ultrasound Imaging

**Fig. 1:**
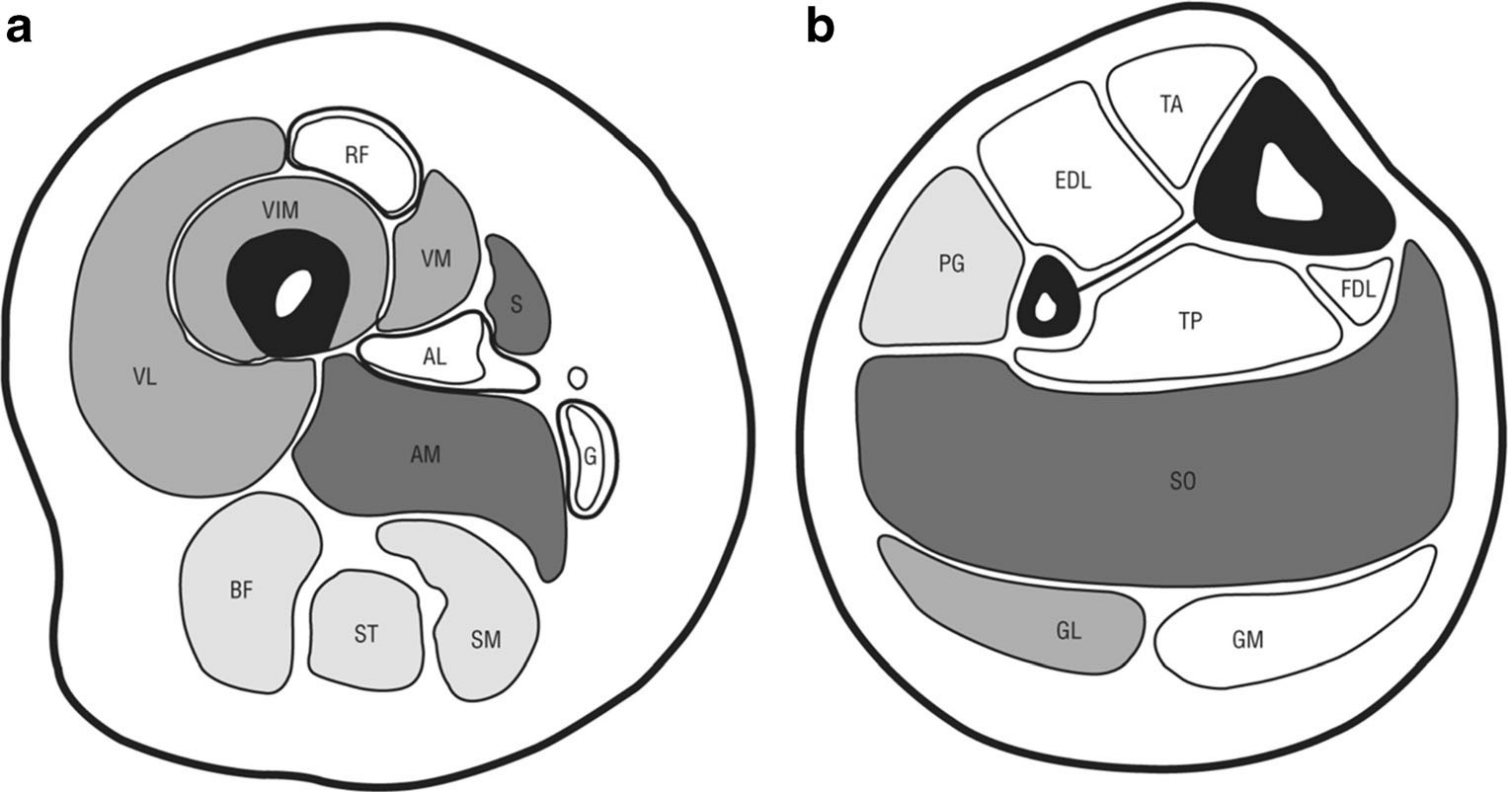
Schematic diagram of the typical pattern in *RYR1-*related myopathies. (**A**) In the thighs, the rectus femoris (RF), adductor longus (AL), and gracilis (G) are spared and in some patients hypertrophied; the adductor magnus (AM), sartorius (S), vastus lateralis (VL), vastus intermedius (VIM), and vastus medialis (VM) are affected; the hamstrings are less affected; and the involvement of semimembranosus (SM) and semitendinosus (ST) is nonspecific. BF = biceps femoris. (**B**) In the calf, the most affected muscle is the soleus (SO), followed by the gastrocnemius lateralis (GL) and to a lesser effect the gastrocnemius medialis (GM). In the anterior compartment, which is less affected than the posterior, the peroneal group (PG) is more affected than the tibialis anterior (TA). EDL = extensor digitorum longus; FDL = flexor digitorum longus; TP = tibialis posterior. (From: Klein et al., 2011, *JAMA Neurology*, with permission)

Muscle magnetic resonance imaging (MRI) and muscle ultrasound imaging can reveal patterns of involvement or sparing of specific muscles in *RYR1*-RM. It is important to recognize, however, that there is overlap among subtypes of congenital myopathies, for example, relative sparing of vastus lateralis in CM resulting from variants in *RYR1*, *SEPN1*, *MYH7*, and *BIN1* [85]. In the upper leg of CCD-dominant cases (Fig. 1), there is fatty infiltration and muscle wasting of the vasti, adductor magnus, and sartorius, while the adductor longus, gracilis, and rectus femoris muscles are relatively spared. In the lower leg, the most affected muscle is the soleus, followed by the lateral head of the gastrocnemius, with selective sparing of the medial head of the gastrocnemius. In the anterior compartment muscles of the calf, the peroneal group is more affected than the tibialis anterior. There is selective involvement in bicep brachii, subscapularis, lumbar paravertebral, and glutei muscles [18, 86]. Recessively inherited *RYR1*-RM subtypes, other than CCD, typically show more diffuse muscle involvement rather than the classic pattern above [30].

## *RYR1*-RM Therapeutic Approaches

### Preclinical Models

In the preclinical setting, investigation of potential therapeutic candidates for *RYR1*-RM is undertaken by utilizing models of the disease (*in vitro*, *ex vivo*, and *in vivo*). Patient biopsy-derived, cultured mature skeletal muscle cells (myotubes) are an ideal *in vitro* model of the disease through which to test potential therapies that target a specific variant or a shared disease pathomechanism. Patient-derived fibroblasts (obtained through skin biopsy or skeletal muscle primary culture) should be retained as such cells can undergo *MyoD* transfection to achieve a myogenic phenotype [87]. Yet, when skeletal muscle tissue is not available from a patient, unaffected immortalized cells (e.g., lymphoblastoid cells, HEK-293 cells which do not have endogenous RyR) represent a stable model through which to recapitulate the disease phenotype by transiently transfecting mutant RyR1 cDNA [88].

Two of the 4 *RYR1* mouse models have been primarily used to test therapeutic candidates for *RYR1*-RM *in vivo.* The I4895T knock-in mouse model exhibits a progressively severe clinical phenotype associated with decreased RyR1-mediated Ca^2+^ permeation and may therefore not be directly comparable to the human disease process resulting from the equivalent variant (I4898T) [47]. In contrast, the Y524S knock-in mouse (corresponding to Y522S in humans) exhibits a mild phenotype with elevated RyR1-mediated Ca^2+^ leak and is comparable to rhabdomyolysis and MH susceptibility clinical phenotypes in humans [51]. A homozygous and RyR1-null, dyspedic mouse model has also been established [89]. Although dyspedic mice die at birth owing to asphyxia resulting from skeletal muscle paralysis, this model has been successfully used to investigate components of EC coupling [90]. An MH mouse model had also been developed with the p.Arg163Cys *RYR1* variant. In the homozygous state, the variant is embryonically lethal whereas the heterozygous form survives and exhibits halothane hypersensitivity [91]. However, there is no histopathology associated with this model [92], suggesting it is a valuable tool for researching MH but perhaps less so for *RYR1*-RM.

Alternatively, a severe homozygous zebrafish *RYR1*-RM model (*relatively relaxed*) has been established which resembles *RYR1*-related MmD [93]. This has proven useful for conducting high-throughput drug screening, and studies, using this model, provided rationale for the first *RYR1*-RM clinical trial [50]. High-throughput drug screening, especially using the *relatively relaxed* zebrafish model but also other techniques, such as assessing calcium ER/SR stress using genetically encoded biosensors *in vitro* [94, 95], has recently enabled identification of several candidate therapeutic compounds, signifying that the era of therapeutics for *RYR1*-RM is advancing at a more rapid pace than before.

Nevertheless, collectively, there is no ideal *in vivo RYR1*-RM animal model, but work is underway to develop a model that better resembles this spectrum of diseases as a whole. Caution must also be taken when comparing results across different model systems [26].

### Therapies

Despite an unmet medical need, there remain no approved therapies for *RYR1*-RM and those discussed in this section are, at best, in investigational stages. Therapies currently under development can be broadly assigned into 2 categories: 1) drugs with a mechanism of action that may negate the downstream deleterious effects of oxidative and nitrosative stress on the cellular milieu and RyR1 posttranslational modifications or 2) drugs that act either directly on RyR1 itself or modulate interacting proteins that determine RyR1 functionality.

**Fig. 2:**
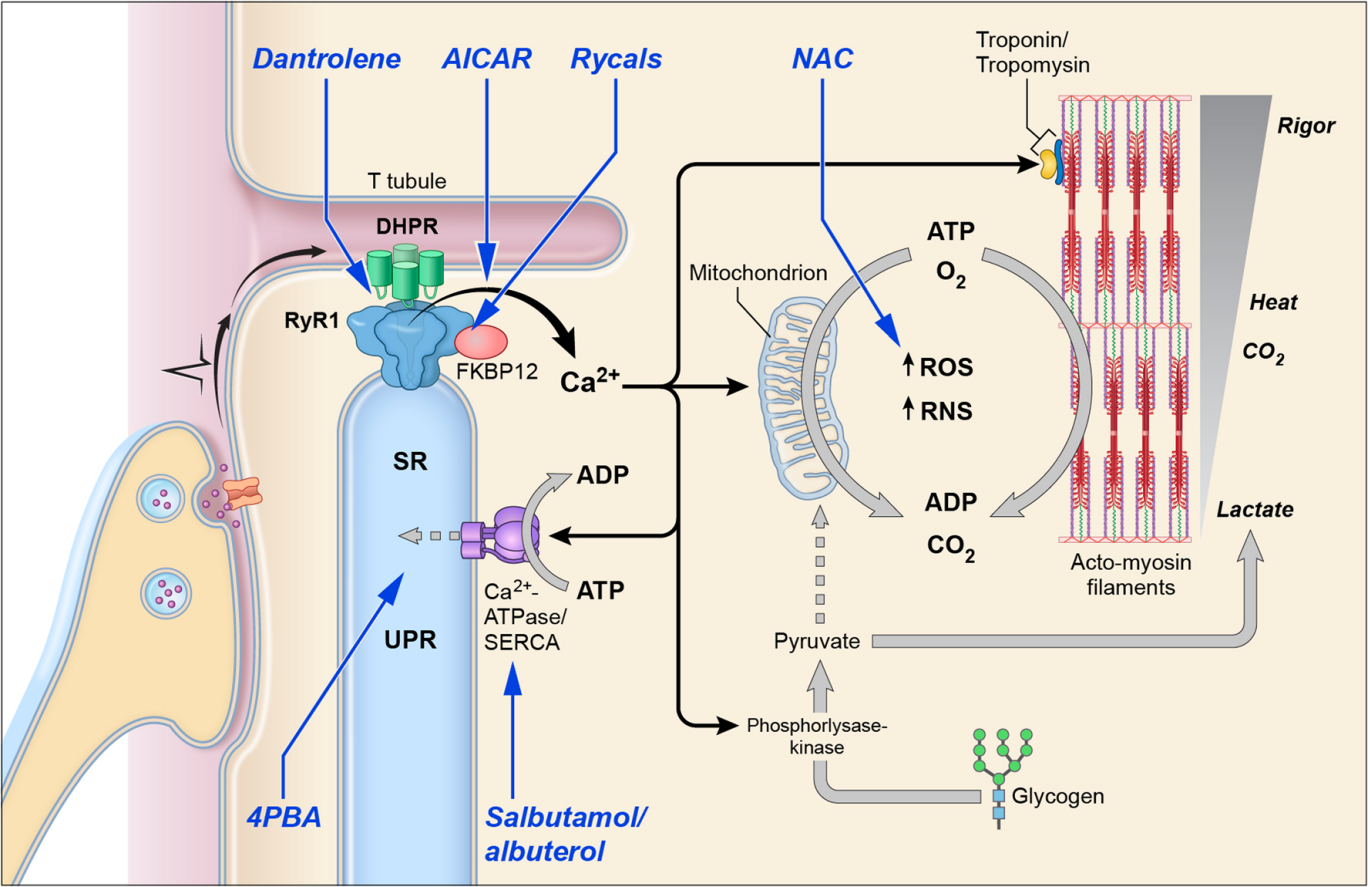
RyR1 channel in the open and closed state. Purported mechanisms of action of various therapies: salbutamol increases SERCA expression levels to facilitate reuptake of cytosolic Ca^2+^ into the SR lumen; NAC works in the sarcoplasm to reduce levels of mitochondrially derived oxidative stress via restoring redox balance; rycals increase FKBP12 binding to RyR1, which in turn maintains the RyR1 channel in the closed state, reducing Ca^2+^ leak into the sarcoplasm; dantrolene antagonizes the RyR1 channel and thus reduces Ca^2+^ leak; 4PBA reduces ER stress markers/UPR and cytosolic Ca^2+^ levels and reduces calpain activation while increasing SR Ca^2+^ content. (Adapted from Witherspoon and Meilleur, 2017 and https://www.biozentrum.uni-wuerzburg.de/humangenetik/forschung/emeritus/prof-mueller-reible/ with permission from Dr. M. Anetseder and Dr. A. Hoyer, Dept. of Anesthesiology, University of Wuerzburg, Germany)

*RYR1* exceeds 159 kb and thus far supersedes the current packaging capacity for adenovirus-mediated therapy (∼ 5 kb) [96]. As such, gene therapy approaches, such as those that have shown great promise for spinal muscular atrophy [97], are not currently feasible for *RYR1*-RM. An additional factor complicating this approach is that more than 700 variants have been located throughout the *RYR1* coding region and canonical splice sites. Although not suitable for all cases, exon-skipping has shown promise, *in vitro*, as a gene-directed therapeutic strategy for compound heterozygous *RYR1*-RM-affected individuals with pseudo-exon inclusion [98]. Despite the abovementioned limitations, at least 3 nongenetic medications have been used, off label, to manage *RYR1*-RM-related symptoms. These include dantrolene, salbutamol/albuterol, and N-acetylcysteine (NAC) (Table 2). The current state of nongenetic medications and therapies under development for *RYR1*-RM are detailed herein with an overview of proposed mechanism(s) of action provided in Fig. 2.

**Table 2:** Compounds under development as potential therapeutics for *RYR1*-RM

Compound/drug	Mechanism of action	Stage of clinical development
N-Acetylcysteine (NAC)	Reduction of aberrant oxidative stress	Phase I/II clinical trial data collection complete, data analysis pending, FDA approved for other indications
Rycal ® (S48168)	RyR1 closed channel stabilization	Phase I complete, phase IIa pending
Sodium 4-phenylbutyrate (4BPA)	Chemical chaperone	Preclinical, FDA approved for other indications
5-Aminoimidazole-4-carboxamide ribonucleoside (AICAR)	RyR1 channel antagonist	Preclinical
Salbutamol/albuterol	Enhancement of SERCA expression	Open-label study complete, FDA approved for other indications
Dantrolene	RyR1 channel antagonist	FDA-approved medical antidote for MH crises, but no formal analyses in *RYR1*-RM
Carvedilol	Beta-blocker	Preclinical, FDA approved for other indications
Pyridostigmine	Acetylcholinesterase inhibitor	Case report of initial response in myasthenic-like presentation, FDA approved for other indications

### Therapies Targeting Downstream Pathomechanisms

Elevated oxidative stress is a hallmark pathological feature in *RYR1*-RM and has been identified in several preclinical models of the disease [47, 50, 51, 99]. In particular, lipid peroxidation of mitochondrial membranes has been implicated as a central pathomechanism that leads to severe mitochondrial dysfunction and/or death in the RYR1^Y522S/W^ mouse model [51]. Owing to elevated mitochondrial Ca^2+^ uptake in response to RyR1-mediated SR Ca^2+^ leak, respiratory chain activity is thought to be upregulated. Resultant reactive byproducts, such as reactive oxygen species (ROS) and nitrogen species (RNS), trigger posttranslational modifications of RyR1 resulting in further calcium dysregulation [100–102].

#### NAC

NAC is a precursor to the ubiquitous antioxidant glutathione and is available in 3 formulations: intravenous (IV), inhaled, and oral. Upon administration, NAC is enzymatically de-acetylated to form cysteine, the rate-limiting component of reduced glutathione resynthesis [103, 104]. The oral formulation is available over the counter, and the intravenous and inhaled formulations are FDA approved for multiple conditions, including acetaminophen overdose and various pulmonary conditions [104–109]. Strong preclinical evidence for a beneficial effect of NAC treatment on skeletal muscle function and histology has been provided by studies conducted in mouse and zebrafish models of the disease. Notably, treatment of the RYR1^Y522S/W^ mice with N-acetylcysteine (NAC) (ad lib via drinking water for 8 weeks) rescued lipid peroxidation and concomitantly prevented previously observed decrements in force generation [51]. These findings have recently been supported by a longer-term study of NAC treatment in the same mouse model in which NAC treatment led to a decreased number of structural cores and improved skeletal muscle function [99]. Further preclinical evidence supporting the therapeutic potential of NAC was provided by Dowling and colleagues (2012) [50]. In an elegant study conducted in *relatively relaxed* zebrafish and *RYR1*-RM patient myotubes, NAC was found to improve endurance and decrease biomarkers of mitochondrial oxidative stress, respectively, when compared to wildtype/control. A double-blind, placebo-controlled trial of NAC (NCT02362425) was recently completed at the National Institutes of Health by our team, and results are pending.

Medical considerations: The dose of NAC required to elicit a potential beneficial effect in this population may be greater than that used OTC for other purposes, possibly owing to decreased basal GSH:GSSG ratio in *RYR1*-RM individuals compared to the general population. Therefore, although available OTC, affected individuals should use NAC under the care of their local neurologist or clinician, and dosing should be weight based.

#### Salbutamol/Albuterol

Salbutamol is a beta 2-adrenergic agonist, typically used for asthma control, but purported to build muscle volume and increase strength. Salbutamol has been reported to increase the overall expression of SERCA (type 1/fast-twitch isoform), a Ca^2+^ ATPase pump responsible for SR Ca^2+^ reuptake although potential mechanisms of action require further investigation [110]. Increased cytosolic Ca^2+^ is a well-established consequence of certain, gain-of-function *RYR1* variants, such as p.Arg4861His and p.Asp4505His, that cause RyR1-mediated Ca^2+^ leak. Subsequently, increased cytosolic Ca^2+^ causes a negative feedback loop to the mitochondria resulting in elevated oxidative and nitrosative stress and possibly poor ATP production.

This drug has been tested in *RYR1*-RM-affected individuals in 2 studies in the past 14 years. The first was an open-label pilot study conducted in London, UK, and included *RYR1*-RM*-*affected individuals with CCD or MmD histopathology [111]. *RYR1*-RM-affected individuals improved in Medical Research Council (MRC) scores, forced vital capacity (FVC), and myometry values [111]. The second report of albuterol treatment involved a single 9-year-old male case with recessive inheritance and purported mitochondrial dysfunction [37]. Treatment led to an improvement in FEV_1_, motor function scales, and clinical observations (such as ability to raise arms above shoulders, speech, and self-care). In both studies, the participants underwent an exercise regimen while taking this medication as it had been alluded, in previous neuromuscular disease intervention, that a combinational effect of albuterol with exercise may exist [112, 113]. Of note, individuals with several neuromuscular diseases anecdotally report improvements with exercise alone; however, exercise as an intervention has not been tested independently in a blinded fashion. Ideally, salbutamol needs to be tested in a randomized controlled trial.

Medical considerations: Given the effect of beta-adrenergics such as salbutamol on cardiac function, an electrocardiogram should be undertaken prior to dosing, in order to rule out long QT syndrome, and monitoring of cardiac rhythm is recommended during administration. This was performed regularly in the case report above via vital signs and periodic electrocardiograms [37].

#### Sodium 4-Phenylbutyrate

Sodium 4-phenylbutyrate (4PBA) is a chemical chaperone and is FDA approved for the management of hyperammonemia due to urea cycle disorders and alleviates endoplasmic reticulum (ER)/sarcoplasmic reticulum (SR) stress. Its mechanism of action is to aid in protein folding, decrease protein aggregation, and lower oxidative stress [47].

A recent study by Lee et al. (2017), using the I4895T *RYR1*-RM mouse model, sought to determine if alleviation of endoplasmic reticulum (ER) stress, uncoupled protein response (UPR), and oxidative stress with 4PBA would reverse skeletal muscle pathology [47]. 4PBA treatment of mice carrying the I4895T *RYR1* variant decreased markers of mitochondrial oxidative stress, muscle protein ubiquitination, and ER stress/UPR and improved skeletal muscle function. Yet 4PBA treatment did not restore SR Ca^2+^ transients in I4895T fibers to wild-type levels, suggesting that decreased SR Ca^2+^ release may not be the central pathomechanism in *RYR1*-RM resulting from this specific variant. Overall, these findings indicate that 4PBA has potential to be repurposed as a therapeutic intervention for *RYR1*-RM patients, at least for those affected by the equivalent human variant (p.Ile4898Thr).

In support of the therapeutic potential of 4PBA, Ma et al. reported decreased muscle wasting in rats with severe burn injuries, following treatment [114]. ER stress markers were elevated in these rats compared with those in the placebo group, especially at postburn days 4 and 7. 4PBA treatment decreased ER swelling, ER stress markers, cytosolic Ca^2+^ concentration, and calpain activation while increasing SR Ca^2+^ content. 4PBA treatment also improved muscle structure and decreased skeletal muscle wasting.

### Therapies Targeting the RyR1 or Modulating Interacting Proteins

#### Dantrolene

Dantrolene is a muscle relaxant and is approved for use as the only medical treatment for an active malignant hyperthermia episode. Although the mechanism of action for dantrolene is not completely understood, this postsynaptic muscle relaxant likely interferes with SR Ca^2+^ release via disruption of communication between RyR1 and Ca_v_1.1 [115]. By inhibiting SR Ca^2+^ efflux, it decreases excitation–contraction coupling and relaxes cardiac and skeletal muscle [116]. After its implementation for treatment of MH, mortality due to MH decreased from 80 to 10% [117].

Reports of off-label dantrolene use for management of *RYR1*-RM symptoms have yielded mixed findings. For example, in a single case with mild CCD, motor function improved [118]. Anecdotally, affected individuals who have used dantrolene off-label state it improves symptoms such as myalgia; however, despite the likely overlap between MH, myalgias, and exertional rhabdomyolysis, further studies are needed to identify the efficacy of dantrolene in these latter 2 phenotypes [36]. Another concern with dantrolene use for *RYR1*-RM is that, given an inhibitory mechanism of action on RyR1, use by affected individuals with variants that already cause decreased sensitivity to RyR1 agonists may result in deleterious sequelae. Although motor function improved in the above case, muscle weakness from dantrolene use has also been reported [36]. *RYR1* variants associated with decreased sensitivity to agonists (also termed “blocked channel variants”) include p.Ile4898Thr, which has been recapitulated in the I4895T mouse model and exhibits decreased resting cytosolic Ca^2+^ and an uncoupled protein response. However, studies of patient-derived myotubes with the corresponding human mutation, p.Ile4898Thr, show a differing phenotype of minimal leak. To this point, Marty and Faure discuss the difficulty of identifying exact pathophysiologic mechanisms while differing preclinical models are used for the same variant, and they consequently surmise that actual insults are likely more complex than our current understanding [26].

Medical considerations: Although it is plausible that affected individuals with variant(s) associated with RyR1 Ca^2+^ leak may benefit from periodic dantrolene use, confirmation of channel leakiness may be difficult for a given variant. More preclinical research is necessary to confirm the functional impact of specific *RYR1* variants, and likely impact of cytosolic free magnesium concentrations on dantrolene action in this population [119, 120], in order to determine the safety and utility of dantrolene administration [36] with the potential for a future personalized health approach in the clinic.

#### Rycals

Rycals are a class of benzothiazepine-derived Ca^2+^ channel stabilizers that have been developed by ARMGO Pharma Inc. (Tarrytown, NY) for chronic heart failure, cardiac arrhythmia, and catecholaminergic polymorphic ventricular tachycardia [121]. Rycals target the interaction between RyR and calstabin isoforms (RyR1 to FKBP12 (calstabin1) in skeletal muscle and RyR2 to FKBP12.6 in cardiac muscle), the latter of which stabilize the RyR closed state under normal physiological conditions without perturbing functional isoforms expressed elsewhere [122]. In skeletal muscle, each RyR1 monomer houses a single FKBP12 binding site localized to the cytosolic shell domain. Preclinical work in skeletal muscle using Rycal compounds has focused on muscular dystrophies, which are considered to have a secondary defect in EC coupling. Animal studies [123], particularly those conducted in the *mdx* mouse model of Duchenne muscular dystrophy [124], have revealed that Rycal compounds restore RyR1-calstabin1 binding, an interaction which is diminished in muscle diseases associated with excessive oxidative and/or nitrosative stress [125]. Under such conditions, Rycal treatment has been demonstrated to restore RyR1-calstabin1 binding thereby stabilizing RyR1 in the closed state [124, 126, 127]. Owing to therapeutic potential, ARMGO Pharma Inc. have expanded their clinical testing pipeline to encompass Rycal treatment for indications related to skeletal muscle diseases, including *RYR1*-RM.

The Rycal class of drugs emerged from initial work using K201 (JTV-519), a compound first described in the early 1990s [128] that has shown mixed effects on RyR1-mediated Ca2^+^ release in vitro and may diminish SR Ca^2+^ re-uptake via SERCA [129]. Owing to this nonspecific mechanism of action, a number of refined JTV-519-derivatives have since been developed including the first Rycal compound to be tested clinically (ARM036; Aladorian), and the current lead Rycal compound (ARM210; S48168). First-in-man phase 1 trials for ARM210 have recently been completed; however, to date, there are no published reports of Rycal treatment in preclinical models of *RYR1*-RM. To address this, using skeletal muscle obtained from *RYR1*-RM-affected individuals, Rycal treatment is currently being tested *ex vivo* through a collaboration between our laboratory and Marks and colleagues at Columbia University. Due to the promising safety profile and therapeutic potential of ARM210, by addressing RyR1-mediated Ca^2+^ leak, a central pathomechanism associated with *RYR1*-RM, a phase 1 trial in this population is in planning stages. In addition, in August 2018, the FDA awarded ARM210 Orphan Drug Designation for *RYR1*-RM.

#### Carvedilol

Carvedilol, a beta-blocker commonly used to treat and manage heart failure and cardiac arrhythmias, suppresses store overload-induced calcium release (SOICR) in HEK-293 cells expressing MH/CCD-associated *RYR1* variants in different regions of the RyR1 channel, including leaky N-terminal variants [130]. SOICR is a depolarization-independent Ca^2+^ overload-induced SR Ca^2+^ release that result in spontaneous muscle contractions [131]. Although the molecular mechanisms underlying the suppression of enhanced SOICR remain undetermined, carvedilol is hypothesized to suppress the RyR1 open state-dependent luminal Ca^2+^ activation and SOICR by stabilizing the closed state of the RyR1 channel [132]. As HEK-293 cells lack certain muscle-specific proteins (L-type Ca^2+^ channels, calsequestrin, triadin, junction), these findings await demonstration in skeletal muscle cells. Carvedilol may represent a potential long-term therapeutic agent for MH and other *RYR1*-RM if future studies show similar action in skeletal muscle [130].

#### 5-Aminoimidazole-4-Carboxamide Ribonucleoside

5-Aminoimidazole-4-carboxamide ribonucleoside (AICAR) has been shown to improve muscle endurance without exercise by activating AMPK, a kinase and cellular energy sensor. Its activation depends on increases in AMP:ATP. Lanner et al. demonstrated that AICAR significantly decreased Ca^2+^ leak and in turn, ROS and RNS generation in the Y524S mouse model [133]. Death typically occurs following heat exposure in this mouse model; however, this was prevented with AICAR treatment, thus indicating that AICAR may be beneficial, prophylactically, in those with exercise or heat intolerance due to variants in *RYR1*. AICAR has also shown benefit in aged myostatin knockout mice, improving running time and peroxisome proliferator-activated receptor-gamma coactivator (PGC)-1alpha expression. To some degree, AICAR also improved mitochondrial function, although it did not increase the number of mitochondria. These benefits were either not observed or not observed to the same degree in aged wild-type mice [134]. Mitochondrial oxidative stress is associated with elevated cytosolic Ca^2+^ levels and has been implicated as a downstream pathomechanism in preclinical models of *RYR1*-RM [50, 135].

#### Pyridostigmine

Pyridostigmine, an acetylcholinesterase inhibitor, has been reported to improve fatigability related to neuromuscular junction (NMJ) signaling in CNM-*RYR1*-related cases [36]. Structural changes (reduced synaptic vesicles; short, shallow, and unbranched synaptic clefts) and abnormal innervation at the NMJ have been correlated with muscle disuse or motor endplate immaturity in CNM [136]. Additionally, decreased quantal release, reduced postsynaptic response, and acetylcholine receptor deficiency compromise neuromuscular transmission at the NMJ in CNM [137]. Response of *RYR1*-RM fatigable weakness to NMJ therapy such as pyridostigmine has been beneficial but nonsustained and therefore awaits further systematic confirmation [136].

### Future Directions

#### Drug Development Considerations

The time taken to reach a definitive *RYR1*-RM diagnosis has been bolstered by NGS advances. However, the lag in bringing therapeutic compounds to clinical trial is recognized as a shortfall that needs addressing for rare diseases as a whole. For example, despite being successfully tested in an open-label study 14 years ago, salbutamol has not been tested in a randomized controlled trial and is still not officially approved for *RYR1*-RM-affected individuals.

This can present a financial burden for affected individuals, whose insurance may or may not cover off-label drugs in certain countries such as the USA. Indeed, passing of the Orphan Drug Act (ODA) in the USA paved the way for the FDA to establish “Orphan Drug Designation” which provides drug development incentives for sponsors working in the rare disease field. Despite this milestone, the drug development timeline for rare disease indications remains slow, in part owing to a backlog of ODA requests and, in 2017, the FDA unveiled a strategic plan to streamline the process. The resources, expertise, and sites available to perform simultaneous clinical trials remain a critical need and cannot be underestimated. In fact, a NAC trial in the *SEPN1*-RM population has been put on extended hiatus due to lack of continued resources, a common problem [138].

While advancing novel compound development is important for advancing potential *RYR1*-RM therapeutics, focus should also be placed on repurposing drugs already approved by regulatory bodies, such as the FDA, for other indications. The latter approach may represent a faster path to *RYR1*-RM (or congenital myopathy) orphan drug designation because such drugs have an established safety profile, and therefore, phase I trials may not be required.

#### Combinatorial Therapy

Future clinical trials may need to involve more complex designs with more than 2 arms in order to assess the potential benefits of combinational therapeutic approaches. A traditional parallel-group RCT with 2 arms (treatment and placebo) may need to be supplemented by adding an arm combining 2 treatments to test for their synergistic effect. Factorial RCT or crossover designs (with washout) may be helpful to this effect. As in various other conditions, future approved treatments may ultimately involve more than one compound, or a “cocktail,” to address different angles of the pathomechanism of the disease [127].

#### Clinical Trial Readiness

As advances in NGS technology improve genetic testing methodology, the likelihood of identifying causative variants in diseases such as *RYR1*-RM is increasing. The trend towards using molecular genetic testing as the first diagnostic tool in CM, without muscle biopsy, is gaining momentum in the field of neuromuscular diseases. This genetics-led approach is justified based on the abovementioned histopathological overlap and its time- and cost-effective nature. By proxy, detailed genetic characterization of *RYR1*-RM-affected individuals will aid in optimizing the specificity of inclusion criteria for clinical trials. As such, clinical trial teams will be able to take into account the drug’s mechanism of action in the context of each individual’s genotype, as well as expected structural modifications, and/or functional consequences.

Accordingly, personalized therapeutic approaches may be required to adequately address the deleterious effects of specific variants and alleviate affected individuals’ symptoms. Emphasis should be placed on developing infrastructure that supports such approaches as it is highly unlikely that a “one treatment suits all” approach will be successful for this population.

## Conclusion

The heterogeneous and expanding spectrum of *RYR1*-RM reflects the implication of calcium dysregulation on fundamental processes in the skeletal muscle cell. Presently, drugs approved for alternative indications have been used to manage symptoms in affected individuals, reflecting a palpable need for additional clinical trials in this population. However, securing sites to perform this labor-intensive work remains challenging, especially given the obstacles of performing trials in rare diseases. Current clinical trial efforts, further understanding of the structure and function of RyR1, and the role of specific pathogenic *RYR1* variants will provide a path forward to investigating effective therapeutic agents to manage and treat *RYR1*-RM.
